# Octreotide Causing Hyperkalemia: A Case Report and Review of the Literature

**DOI:** 10.7759/cureus.68246

**Published:** 2024-08-30

**Authors:** Sandeep Sasidharan, Sabu John, Isha Puri, Muhammad Azhar, Mary Mallappallil

**Affiliations:** 1 Nephrology, State University of New York (SUNY) Downstate Health Sciences University, New York City, USA; 2 Nephrology, New York City (NYC) Health + Hospitals/Kings County Hospital Center, New York City, USA; 3 Cardiology, New York City (NYC) Health + Hospitals/Kings County Hospital Center, New York City, USA; 4 Cardiology, State University of New York (SUNY) Downstate Health Sciences University, New York City, USA

**Keywords:** sulphonylurea oral hypoglycemic, medical icu, dextrose, ekg abnormalities, recurrent hypoglycemia, drug induced hyperkalemia, octreotide

## Abstract

Octreotide, a synthetic analog of somatostatin, is widely utilized for its inhibitory effects on various hormones, including growth hormone, insulin, and glucagon. Its applications span conditions such as acromegaly, carcinoid tumors, and gastrointestinal bleeding due to its ability to reduce portal venous pressure. Additionally, it serves a crucial role in nuclear medicine imaging and the management of hepatorenal syndrome. We report a case of a 44-year-old man with type 2 diabetes mellitus (T2DM) and stage 5 chronic kidney disease (CKD), not yet on hemodialysis, who presented with persistent severe hypoglycemia. Despite multiple oral glucose administrations, his blood glucose levels remained critically low. The patient was treated with octreotide for sulfonylurea-induced hypoglycemia. However, he developed hyperkalemia as a side effect of octreotide treatment. Traditional therapies for sulfonylurea-induced hypoglycemia often involve intravenous and oral dextrose and glucagon, which may lead to recurrent hypoglycemia due to their stimulatory effects on insulin release. Octreotide directly inhibits insulin release from the pancreas, thus preventing rebound hypoglycemia. However, its administration in patients with renal impairment poses a risk of hyperkalemia due to its suppression of insulin-mediated cellular potassium uptake, and it should be used with caution. This case highlights the potential for life-threatening hyperkalemia induced by octreotide in non-dialysis CKD patients. Physicians must be vigilant about this side effect, particularly in patients with underlying renal impairment. Close monitoring of potassium levels and appropriate management strategies are essential to ensure the safe use of octreotide. This case aims to raise awareness and contribute to a better understanding of octreotide-induced hyperkalemia in CKD patients.

## Introduction

Octreotide, a synthetic analog of somatostatin, is a peptide medication that is used for its somatostatin-like properties, which include inhibition of growth hormone, insulin, and glucagon [1]. It exerts its pharmacological effects by binding to somatostatin receptors, which are found in the pancreas, gastrointestinal tract, and central nervous system. By doing so, it inhibits the release of various hormones, such as growth hormone, insulin, glucagon, and gastrointestinal peptides.

Octreotide is a more potent inhibitor of these hormones, and it is used in conditions like acromegaly [2], carcinoid, and vasoactive intestinal peptide-secreting tumors, and by reducing portal venous pressure, it acts as an effective treatment in gastrointestinal bleeding [3]. Further, it is used in nuclear medicine imaging to image tumors and tissue that express somatostatin receptors. It is frequently used in hepato-renal syndrome combined with midodrine to reverse peripheral dilation and increase renal blood flow [4].

## Case presentation

A 44-year-old man with type 2 diabetes mellitus (T2DM) and chronic kidney disease (CKD) stage 5, not yet on hemodialysis (HD), presented with complaints of feeling weak and nervous with blood sugars (BS) ranging from 20-45 mg/dL for two days that did not resolve with oral glucose administration. On his prior admission two weeks ago, he had presented with severe hypoglycemic symptoms, including loss of consciousness, diaphoresis, and hypoglycemia, with a blood glucose of 30 mg/dL while on semaglutide and glipizide. With the normalization of serum glucose over that hospital stay, he was discharged and chose to continue glipizide. Other medical history included obesity with a body mass index of 48 kg/m^2^, hypertension, obstructive sleep apnea, and hyperlipidemia. His medications included amlodipine, aspirin, ergocalciferol, simvastatin, valsartan, hydrochlorothiazide, and glipizide.

On physical examination, blood pressure was 138/80 mmHg, heart rate was 93 bpm, temperature was 97.8 F, respiratory rate was 21/min, and oxygen saturation was 85% on room air, which improved to 96% on 3 L provided via nasal cannula. His physical examination showed an obese individual with bilateral lower extremity edema. The rest of the examination was within normal limits. Laboratory test results are shown in Table 1.

**Table 1:** Laboratory data BMP: Basic metabolic panel; CBC: Complete blood count; VBG: Venous blood gas; eGFR: Estimated glomerular filtration rate; K: Potassium; pCO_2_: Partial pressure of carbon dioxide; pO_2_: Partial pressure of oxygen; HCO_3_: Bicarbonate

		On day of the presentation	After octreotide treatment	After hyperkalemia treatment	After hyperkalemia treatment with dialysis
BMP	Normal range				
Sodium (mmol/L)	136-147	145	140	138	141
Potassium (mmol/L)	3.5-5.3	5.6	7.3	6.7	4.7
Chloride (mmol/L)	100-112	107	103	102	102
Bicarbonate (mmol/L)	19-29	23	26	25	33
Blood urea nitrogen (mg/dL)	6-24	52	49	48	40
Creatinine (mg/dL)	0.5-1.4	8.06	7.34	7.13	5.78
Blood glucose (mg/dL)	70-110	45	173	238	116
Calcium (mg/dL)	8.4-10.5	8.4	8.7	8.7	8.4
Albumin (g/dL)	3.3-5.0	3.8	4		
eGFR (ml/min/1.73m^2^)	>60	7.8	8.8	9.1	11.7
Anion gap (mEq/L)	5-15	15	11		
Endocrine					
Insulin (mU/L)		7.05			
CBC					
WBC (K/μL)	3.8-10.5	4.9	4.28		4.67
Hemoglobin (g/dL)	13-17	10.7	11.3		10.2
Hematocrit (%)	39-50%	37.2	40.6		36.1
Platelet count (K/μL)	150-400	183	208		87
VBG					
pH	7.32-7.43	7.24	7.25		7.27
pCO_2_ (mmHg)	41-54	66	67		68
pO_2_ (mmHg)	30-50	48	32		50
K (mmol/L)	3.5-5.3	5.2	7.1		4.4
HCO_3_ (mmol/L)	19-29	30	32		33
Lactate (mmol/L)	0.6-1.4	1.3	1.5		0.7

The chest X-ray showed a stable enlarged cardiac silhouette and unchanged prominent pulmonary vasculature. There was a small left pleural effusion. The ultrasound of the kidneys showed bilaterally increased echogenicity and simple renal cysts.

**Figure 1:**
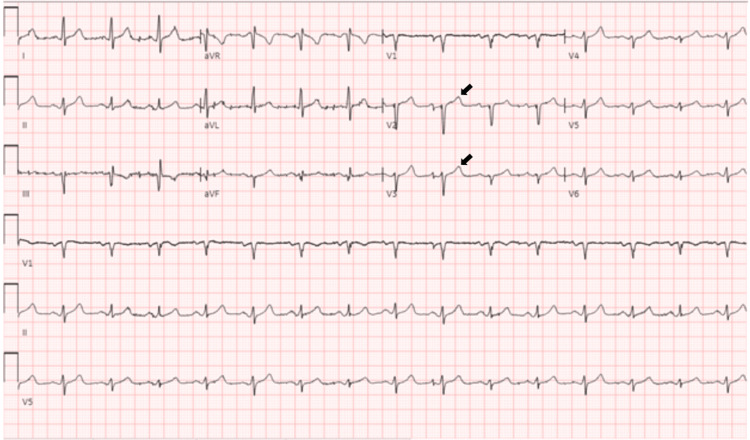
Pre-octreotide electrocardiogram with serum potassium of 5.6 mmol/L. It shows normal sinus rhythm and normal TV amplitude in leads V1 and V2 (marked by arrows).

**Figure 2:**
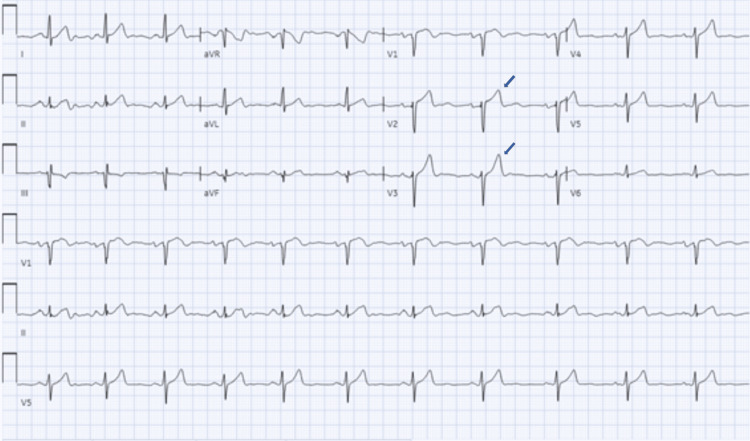
Electrocardiogram after octreotide with serum potassium of 7.3 mmol/L. It shows normal sinus rhythm with peaked T waves in leads V2 and V3 (marked by arrows).

The patient was diagnosed with symptomatic hypoglycemia and acute kidney injury (AKI) on CKD. He needed admission to the intensive care unit (ICU) for persistent hypoglycemia despite multiple ampules of dextrose 50% (D50) and dextrose 10% (D10) continuous intravenous (IV) infusion. In the ICU, he was started on an octreotide bolus and IV infusion. His blood glucose stabilized between 180-220 mg/dL on octreotide but his serum potassium increased to 7.3 mEq/L with electrocardiogram (EKG) changes as shown in Figure 1 and Figure 2.

**Figure 3:**
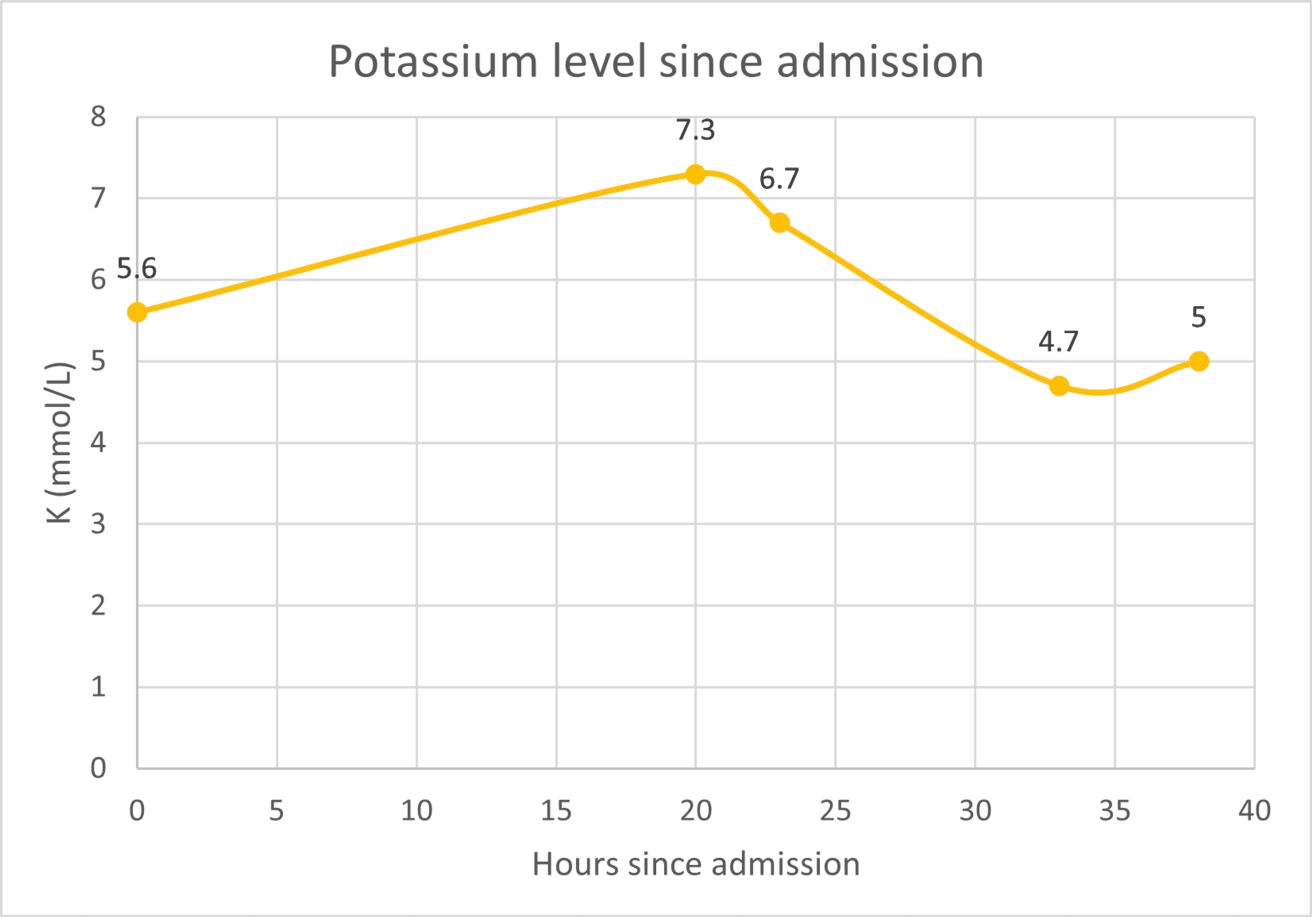
Serum potassium levels since hospitalization K: Potassium

Hyperkalemia was medically treated with D50, regular insulin, sodium zirconium cyclosilicate, albuterol, calcium gluconate, and furosemide, and octreotide was discontinued. Subsequently, the patient had HD using a dialysate with 2 mEq/L potassium and 2.5 mEq/L calcium concentrations. Serum potassium levels improved to the normal range (Figure 3). With his advanced kidney failure, he was started on three times a week maintenance HD with a tunneled dialysis catheter. He was treated with linagliptin for his T2DM, which he continued. The patient subsequently got a deceased donor kidney transplantation and is now on prednisone, tacrolimus, mycophenolate, and semaglutide/weekly. He has no further hypoglycemia and continues to do well.

## Discussion

Conventional treatments for sulfonylurea-induced hypoglycemia involve glucagon and intravenous and oral dextrose combined. Despite this treatment, hypoglycemia often recurs or may last longer as glucose is a potent stimulus for releasing more preformed insulin [5,6]. Prolonged infusions of hypertonic dextrose solutions may cause phlebitis and can result in excessive volume retention. Glucagon increases blood sugar levels at the expense of hepatic glycogen stores and may also exacerbate hyperinsulinemia. Unlike dextrose infusions, octreotide, like somatostatin, directly suppresses the pancreatic production of insulin, preventing rebound hypoglycemia. When treating sulfonylurea-induced hypoglycemia, octreotide is the primary treatment of choice because it prevents insulin secretion and maintains euglycemia when combined with dextrose [6,7].

Hypoglycemia is a significant side effect due to hyperinsulinemia state, especially with renal impairment, and long-acting sulfonylurea is a risk factor [8]. When used to treat sulfonylurea-induced hypoglycemia that is refractory to traditional therapy, octreotide has been shown to be a safe and effective medication, particularly for patients with diabetes and renal failure [1]. There are no definitive trials that have established the dosing needed to treat sulfonylurea-induced hypoglycemia in humans. Current recommendations are to give 50 μg every six hours, which was ordered for our patient [1,9], along with a continuous intravenous infusion of dextrose.

At the beginning of treatment, octreotide has been reported to cause a number of mild and transient side effects, such as pain at the injection site, headache, abdominal pain, nausea, vomiting, diarrhea, or constipation. More severe complications like gallstones are seen with increased frequency. Hypothyroidism, arrhythmia, or gastritis can present with long-term treatment. Hyperkalemia has been described as an adverse reaction to octreotide [10].

The inhibition of insulin release, which reduces cellular potassium absorption and raises extracellular potassium concentration, is most likely the mechanism underlying octreotide-induced hyperkalemia [11]. The mechanism of inhibition is through competitive agonism of the somatostatin receptors SSTR2 and SSTR5 [10]. The membrane sodium-potassium pump (Na+/K+ ATPase) maintains the cell's internal milieu, which, with the hydrolysis of ATP, moves three sodium ions (Na+) out and two potassium ions (K+) into the cell against their concentration gradient. The concentration gradient then provides the driving force for the inward movement and co-transport of amino acids and monosaccharides and the outward counter-transport of protons, calcium, and K+ and contributes to other activities of homeostasis and growth. The hormonal regulation of the Na+/K+ ATPase is an important control point for multiple cell processes.

Insulin stimulates a large increase in the sodium acid exchange transport (Na+/H+), and the sodium influx into the cell then stimulates the membrane Na+/K+ ATPase and, therefore, the serum potassium levels [12]. By inhibiting insulin, octreotide affects the suppression of the cell membrane Na+/K+ ATPase and a resulting increase in serum potassium, resulting in hyperkalemia.

Hyperkalemia can result in EKG findings of peaked T waves, flattened P waves, prolonged PR interval, ST depression, prolonged QRS duration, and sine waves. Our patient's first EKG with a serum K+ of 5.6 mmol/L showed normal sinus rhythm with normal T wave amplitude (Figure 1). The second EKG, which was done after octreotide administration with serum K+ of 7.3 mmol/L, showed normal sinus rhythm with tall, peaked T waves in leads V2, V3, and V4 consistent with hyperkalemia (Figure 2).

In cases of insulinomas, sulfonylurea overdose, and prolonged hyperinsulinemic hypoglycemia in infants, octreotide has been effectively used [13-15]. It is primarily metabolized by the liver and is well tolerated by most patients without serious side effects. Some experts believe that dosage adjustments may be necessary if clearance is reduced due to organ dysfunction [16].

Although the patient had advanced CKD, which could cause hyperkalemia due to reduced impaired renal excretion of potassium, his rise in potassium was temporally related to the octreotide administration. The pronounced hyperkalemia observed was a consequence of the inhibition of insulin release caused by octreotide. Hemodialysis was administered to eliminate potassium from the body due to the ongoing hypoglycemia, which restricted the utilization of insulin to facilitate the movement of potassium into cells. 

In our patient, the common causes of hyperkalemia were ruled out to support the diagnosis, which included ensuring the patient was on a low-potassium diet and no potassium-containing fluids or additional hyperkalemia-inducing medications were administered. Hyperkalemic EKG changes ruled out pseudo-hyperkalemia as a diagnosis; no evidence of metabolic acidosis or increased tissue catabolism, and all chemistry results were from non-lysed specimens.

A review of the literature in PubMed, Medline, and Google Scholar showed that there are few reports of octreotide-induced hyperkalemia, including a study of 38 patients with neuroendocrine tumors on peptide receptor radionucleotide therapy (PRR) with octreotide [17] and abstracts like bradycardia, renal failure, atrioventricular nodal blockade, shock, and hyperkalemia (BRASH) syndrome induced by octreotide [18]. There are only four cases of octreotide-induced hyperkalemia in patients with renal dysfunction - two without end-stage renal disease (ESRD) and two with ESRD. In one patient with ESRD, octreotide was used to prevent severe postprandial hypotension [19], while the other was treated for sulfonylurea-induced hypoglycemia [10]. In the other two cases, patients with CKD, one patient had a pancreatic fistula treated with octreotide [20], and the other patient was on octreotide for chronic diarrhea while on total parenteral nutrition but also received low molecular weight heparin (LMWH) for deep venous thrombosis prophylaxis [21]. None of the patients needed treatment with urgent dialysis. Based on these, it is recommended that octreotide be used cautiously in patients receiving dialysis or having advanced CKD where renal excretion of potassium is limited, even if it may be an effective therapeutic option in refractory sulfonylurea-induced hypoglycemia.

## Conclusions

We suspect that hyperkalemia induced by octreotide may be more prevalent and may be caused by multiple factors, as in our patient. To our knowledge, this is the first case of potentially fatal hyperkalemia in an advanced CKD patient due to octreotide used for glipizide-induced hypoglycemia requiring medication and HD. Since there is a paucity of safety data in CKD patients regarding octreotide use, it should be used cautiously and the potassium levels must be monitored closely. Our case report aims to raise awareness of this potential complication and contribute to a better understanding of octreotide-induced hyperkalemia.

In summary, physicians using octreotide should be vigilant for potential side effects such as hyperkalemia, particularly in patients with underlying renal impairment or those receiving medications that may affect potassium balance. Monitoring of potassium levels and appropriate management strategies are essential to ensure the safe and effective use of octreotide therapy.
